# Predictive Modelling of Postpartum Haemorrhage Using Early Risk Factors: A Comparative Analysis of Statistical and Machine Learning Models

**DOI:** 10.3390/ijerph21050600

**Published:** 2024-05-07

**Authors:** Shannon Holcroft, Innocent Karangwa, Francesca Little, Joelle Behoor, Oliva Bazirete

**Affiliations:** 1 Department of Statistical Sciences, University of Cape Town, Cape Town 7701, South Africa; 2 College of Medicine and Health Sciences, University of Rwanda, Kigali 3296, Rwanda

**Keywords:** postpartum haemorrhage, statistical models, machine learning models, sensitivity, specificity, misclassification rate

## Abstract

Postpartum haemorrhage (PPH) is a significant cause of maternal morbidity and mortality worldwide, particularly in low-resource settings. This study aimed to develop a predictive model for PPH using early risk factors and rank their importance in terms of predictive ability. The dataset was obtained from an observational case–control study in northern Rwanda. Various statistical models and machine learning techniques were evaluated, including logistic regression, logistic regression with elastic-net regularisation, Random Forests, Extremely Randomised Trees, and gradient-boosted trees with XGBoost. The Random Forest model, with an average sensitivity of 80.7%, specificity of 71.3%, and a misclassification rate of 12.19%, outperformed the other models, demonstrating its potential as a reliable tool for predicting PPH. The important predictors identified in this study were haemoglobin level during labour and maternal age. However, there were differences in PPH risk factor importance in different data partitions, highlighting the need for further investigation. These findings contribute to understanding PPH risk factors, highlight the importance of considering different data partitions and implementing cross-validation in predictive modelling, and emphasise the value of identifying the appropriate prediction model for the application. Effective PPH prediction models are essential for improving maternal health outcomes on a global scale. This study provides valuable insights for healthcare providers to develop predictive models for PPH to identify high-risk women and implement targeted interventions.

## 1. Introduction

The historical definition of postpartum haemorrhage (PPH) was based on specific blood loss thresholds after vaginal or caesarean delivery. For vaginal delivery, it was defined as a blood loss of 500mL or more, while for caesarean section, it was defined as a blood loss of over 1000mL [1]. However, in 2017, the American College of Obstetrics and Gynaecology revised this definition. The new definition includes any blood loss exceeding 1000mL accompanied by hypovolemia symptoms within 24 hours post-delivery, regardless of the delivery method. This revision was prompted by the routine underestimation of blood loss during delivery [2]. In global clinical practice, there is a lack of consensus on the definition of PPH [3]. This challenges regional and national comparisons of the prevention and management of PPH [3].

PPH primarily occurs within the first 24 hours after delivery but can also manifest up to 12 weeks post-delivery [4]. Bleeding within the initial 24 hours is referred to as primary PPH, while bleeding occurring between 24 hours and 12 weeks post-delivery is termed secondary PPH [4]. Uterine contractive medication, such as oxytocin, is administered as a first-line treatment to prevent and manage primary PPH [3]. Other pharmaceutical treatments, surgical procedures, and blood transfusion may be advised for further management of post-delivery bleeding [3]. 

Globally, pregnancy-related fatalities pose a significant concern and lead to the premature loss of around 500,000 women each year [5]. Among these fatalities, approximately one-fourth are linked to PPH [6]. Despite an overall reduction in maternal mortality rates [7], PPH remains a significant threat, affecting 1–5% of deliveries worldwide and standing as the leading cause of maternal morbidity and mortality [2]. Additionally, for every PPH-related death, there are at least 10 cases of "maternal near misses," which encompass severe complications such as multiorgan dysfunction, multiple blood transfusions, or peripartum hysterectomy [8]. Therefore, the accurate identification of women at a higher risk of PPH is crucial for improving intervention strategies and reducing maternal deaths and adverse outcomes. 

Individual, socio-cultural, and environmental factors influence the detection and management of PPH in hospital settings [9]. A qualitative study conducted in Nigeria, Kenya, and South Africa highlighted knowledge, beliefs about consequences, beliefs about capabilities, and priorities as barriers to PPH detection and management [9]. Semi-structured interviews with hospital-based healthcare professionals identified variable knowledge of the recommended practices surrounding PPH. The interviewed professionals believed that incorrectly identifying a PPH case would bear negative professional consequences. Lack of self-belief in one’s professional ability to manage PPH was frequently reported. The interviewees also perceived PPH detection and management as a low priority within their hospital environments.

Implementing a clinical decision support system (CDSS) can assist healthcare professionals in navigating the complex factors influencing the detection and management of conditions such as PPH [10]. A CDSS is a set of digital tools designed to improve healthcare delivery by supporting higher-quality medical decision-making [10]. Targeted information and recommendations based on patient characteristics and best practices are supplied to the clinician by the CDSS at the point of care. The primary objective is to enhance healthcare professionals’ clinical knowledge, although a CDSS may leverage data sources that are challenging for the clinician to obtain or interpret. Predictive models are often included as decision-making tools within a CDSS [10]. A predictive model estimating a patient’s risk of PPH based on the patient’s characteristics and clinical observations could therefore form part of a CDSS to support the accurate identification of women at a higher risk of PPH and reduce maternal deaths and adverse outcomes.

The present research aims to develop a predictive model for PPH using early risk factors and to rank the importance of these risk factors in terms of predictive ability. It builds upon an observational case–control study conducted in northern Rwanda in 2021 that utilised the same dataset from Rwanda [8]. The primary objective of the earlier investigation was to identify risk factors for PPH and determine its prevalence, which was found to be 25.5%. This prevalence exceeds the global estimated prevalence of 1–5% of deliveries [2]. The 2021 study employed descriptive statistics to assess the overall prevalence of PPH and utilised inferential statistics, specifically a modified Poisson regression model with robust error variance, to identify risk factors for PPH.

The study considered various risk factors related to social and demographic aspects, pregnancy, obstetrics, and factors during and immediately after childbirth. Significant early risk factors were identified, including having no medical insurance, multiple foetuses, pre-labour bleeding, intrauterine foetal death, and haemoglobin level at labour, all at a 5% significance level. Maternal age, body mass index (BMI), multiparity, and a history of PPH were also found to be statistically significant.

While many of these factors are well-known contributors to PPH, Bazirete et al. [11] emphasised the significance of pre-labour haemorrhage and intrauterine foetal death. They recommended a further examination of these factors in subsequent predictive models for PPH.

Findings from research conducted in various Eastern African countries both support and challenge the conclusions of Bazirete et al. [11]. A cross-sectional study conducted at a university hospital in eastern Ethiopia confirmed the significant roles of maternal age, multiparity, and a history of PPH as risk factors for PPH, utilising logistic regression in their analysis [12]. In contrast, a prospective cohort study from Uganda did not establish a significant association between the risk of PPH and factors such as anaemia during pregnancy, a history of PPH, or multiparity based on their logistic regression analysis [13]. However, it was found that multiple pregnancy constituted a significant risk factor for PPH in the case of vaginal delivery, although this was not a consistent risk factor across all delivery methods.

The variations in findings regarding risk factors underscore the need for further exploration of these divergent outcomes. The discrepancies observed may be attributed to the use of different research methodologies and statistical models, highlighting the importance of considering these factors when interpreting the results.

This study aims to develop an optimal predictive model for PPH using early risk factors. It employs both statistical and machine learning models, each with unique advantages [14]. According to these authors, statistical models assume data are generated by a known stochastic model, allowing for direct modelling. Machine learning (ML) approaches consider the data generation process largely unknown. Statistical models predetermine features and their interactions. ML models offer adaptability and flexibility. Features and their interactions do not need to be specified prior to fitting the model to the observed data. Feature selection and interactions are the result of data-driven processes during model training. The adaptability and flexibility of ML models minimise the impact of incorrectly specifying the data generation process in statistical models.

ML models often outperform statistical models in predictive performance but can be less interpretable. Consequently, ML model specifications and outputs may be poorly reported in research publications, and assessments of ML model validity may be less reliable [15]. Consistent interpretations from ML models are a challenge, and they may be prone to misuse as decision-making tools within a clinical decision support system (CDSS) in a hospital setting. 

A review by Christodoulou et al. [16] examined 71 studies and concluded that ML techniques did not significantly improve predictive performance compared to logistic regression in clinical prediction modelling with binary outcomes. However, ML models outperformed logistic regression in high-bias situations. Venkatesh et al. [17] emphasised the transparency of statistical models, making them more suitable for clinical practice, while hybrid techniques combining statistical modelling and ML methods provide interpretable alternatives. 

Venkatesh et al. [17] conducted a study using data from the United States Consortium for Safe Labor Study to develop predictive models for postpartum haemorrhage (PPH). ML techniques, specifically Random Forests and gradient-boosted trees with XGBoost, exhibited superior discriminative power in predicting PPH compared to statistical models such as logistic regression with/without LASSO regularisation. The study validated the models using phased and multi-site data, further reinforcing their findings.

## 2. Materials and Methods

### 2.1. Dataset

This project utilises a dataset from a study carried out by Bazirete et al. [11] in Rwanda, aimed at identifying risk factors for postpartum haemorrhage (PPH) and determining its prevalence, established at 25.5%. The study was an observational case–control analysis conducted from 1 January 2020 to 30 June 2020 across five health facilities in Rwanda’s Northern Province, focusing on women aged 18 and older who delivered at or beyond 32 weeks’ gestation. This is part of a broader study aimed at developing a risk assessment tool (RATP) and investigating preventative factors for postpartum haemorrhage (PPH). The facilities were selected based on their healthcare performance and location accessibility. It included 430 participants (108 cases and 322 controls), with cases defined by clinical criteria such as blood loss over 500 ml within the first hour post-birth or the need for a blood transfusion. Controls were women from the same facilities who did not experience primary PPH. Data were gathered through structured interviews and reviewing comprehensive medical records to ensure accuracy.

As the goal of this study was to construct the most effective predictive model for early PPH detection, risk factors known upon labour admission or during labour were considered the primary variables of interest for predicting PPH. These variables were selected based on the findings of Bazirete et al. [11], Mesfin et al. [12], and Ononge et al. [13] from research conducted in East African countries. 

However, some pre-labour risk factors were extremely rare within the study population, with an incidence of less than 1.5%. As the classification performance depends on having features that clearly differentiate between the majority and minority classes, it is crucial to evaluate whether a variable of interest contains enough information to achieve this separation [18]. 

As a result, certain variables like uterine anomaly, uterine surgery, gestational diabetes mellitus, polyhydramnios, anticoagulant medication, and severe preeclampsia were not considered for this study due to their low prevalence in the dataset. Their limited presence inhibits a clear separation between cases of PPH and the control group based on these variables. The relationships between these low-prevalence variables and PPH cases would be best explored outside of a predictive modelling context.

The risk factors of postpartum haemorrhage (PH) retained for the analysis included maternal age, haemoglobin level during labour (Haem), body mass index (BMI), multiparity (MP), multiple pregnancy (MU), medical insurance (IN), previous PPH (Pre), pre-labour haemorrhage (AH), and intrauterine foetal death (IFD).

### 2.2. Statistical Analysis

In this study, we utilised two statistical models: logistic regression and penalised logistic regression with elastic-net regularisation. Penalised regression applies a penalty to variables with a high variance, resulting in a reduction in the number of variables in the model and an improvement in prediction quality. Elastic-net regularisation is a combination of L1 and L2 penalty terms weighted by a mixing parameter [19]. The inclusion of the logistic regression model with all features was for comparative purposes to consider the impact of L1 penalty feature selection and L2 penalty regularisation on predictive performance.

In addition, this study used three distinct tree-based ensemble learning techniques to construct predictive models for postpartum haemorrhage (PPH): Random Forests, Extremely Randomised Trees, and gradient-boosted trees utilising the XGBoost library. Tree-based ensembles work by creating a collection of decision trees and then consolidating their predictions [20].

At their core, decision trees are simple models that anticipate outcomes from data observations through recursive binary splits on features within the dataset. The binary splits are determined based on optimising a certain splitting criterion. This process effectively partitions the feature space into unique, non-overlapping predictive sub-regions as per a branching set of decision rules [21]. Each partition results in a node in the decision tree, grouping observations deemed similar as per the decision rule.

Final predictions are made at terminal nodes when the space can no longer be partitioned further, or when a particular stopping condition is met. For regression trees, these predictions are continuous in nature and reflect the mean of the target response for the observations partitioned within the terminal node [20]. Conversely, classification trees predict class probabilities and class labels. Hence, the prediction of PPH cases and their probabilities using tree-based methods would be viewed as a classification problem. Here, all observations within a terminal node are classified according to the majority class of the observations partitioned within that node.

To these authors, tree-based ensemble methods may be considered more readily interpretable than many other machine learning techniques. Every decision rule that is applied to partition the feature space is transparent to the user, including both the features and the values or levels on which recursive binary splits are performed.

Random Forests and Extremely Randomised Trees (ERTs) both train trees on random subsets of features rather than the full feature sets. However, while a Random Forest identifies split points based on a prespecified splitting condition, an ERT selects split points at random. Due to the randomisation of the splits, an ERT is often observed to perform better than a Random Forest in classification problems with noisy data [18]. An ERT’s sampling approach differs from a Random Forest’s bootstrap resampling as trees are trained on random samples drawn without replacement from the full set of training observations [22]. On the other hand, gradient-boosted trees use a stochastic gradient-boosting algorithm to train a tree-based ensemble model by optimising an objective function [23].

Hyperparameter tuning was performed via a grid search. Four hyperparameters were considered for the Random Forest and ERT: the number of random features considered at each split, the number of trees in the ensemble, the minimum size of terminal nodes, and the maximum depth of each tree in the ensemble. The following seven hyperparameters were considered for the gradient-boosted trees with XGBoost: the learning rate, the minimum loss reduction, the number of boosting iterations, the minimum child weight, the maximum tree depth, the proportion of features sub-sampled per tree, and the proportion of training observations sub-sampled per tree.

Data analysis was performed using RStudio [24]. This study employed a 40–60 data separation, with 60% of the data used for training and the remaining 40% for validation (30%) and testing (10%). All candidate models were trained, tested, and validated on the same data separation. As recommended by Mehrnoush et al. [25], while various separations and approaches for training and test data can be utilised, it is crucial to maintain consistent separation across all algorithms for meaningful comparisons.

As classification performance often varies with different training sets, this study identifies the model with the best classification performance across three different data partitions over 5-fold cross-validation. Using this approach allows the analysis to consider the stability and generalisability of the predictive models across different subsets of the data. These data partitions were created using the tidymodels package [26]. To encourage balanced training sets with respect to the target variable (PPH), training set partitioning was stratified by the target variable. Each training set consisted of 24.9% of PPH cases, but the proportions of PPH cases differed in the validation and test sets. Given the sample size (*n* = 430), there would be considerable overlap in the training sets (and thus validation and test sets) if more data partitions were created. Comparing classification performance across overlapping data partitions would be redundant.

All candidate models were trained to maximise the area under the ROC curve and were evaluated using sensitivity to PPH cases and specificity to controls as comparative measures. 

The area under the Receiver Operating Characteristic (ROC) curve is a widely recognised metric in machine learning [27]. The ROC curve plots the true positive rate (TPR) against the false positive rate (FPR) at different probability thresholds. In this application, the ROC curve plots the proportion of correctly predicted PPH cases against the proportion of incorrectly predicted PPH cases. The area under this ROC curve is then used to assess the performance of a classification model at different probability thresholds.

Sensitivity refers to the proportion of true PPH cases accurately predicted by a given model, while specificity denotes the proportion of true PPH controls accurately predicted. 

Candidate models were trained with 5-fold cross-validation using the caret package [28], and probabilities of PPH were predicted. Each candidate model’s sensitivity to PPH cases and specificity to controls were calculated separately for each data partition. The classification probability threshold providing the optimal trade-off between sensitivity and specificity was identified graphically using the area under the ROC curve. The means of these partition-specific metrics were calculated to obtain an average sensitivity and specificity across the three data partitions for each candidate model.

The best-performing predictive model of PPH provides a good balance between both sensitivity and specificity across folds and data partitions. Practical applicability is a further consideration when recommending the best-performing predictive model for medical professionals.

The average misclassification rate across the data partitions was also reported for the final model to provide a practical representation of the model’s predictive performance. The misclassification rate (MR) describes the proportion of all predictions that were incorrectly classified.

A ranking of feature importance in predictors of PPH was obtained from the best-performing predictive model. Considering feature importance across data partitions may provide a ranking that is more stable and generalisable than if a single partition was considered.

## 3. Results

**Figure 1:**
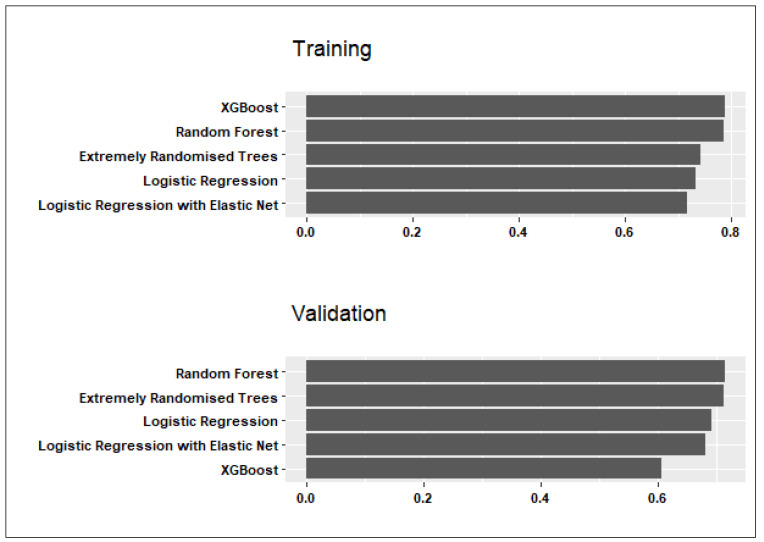
Comparison of candidate model sensitivity averaged across the training sets produced by three different data partitions.

**Figure 2:**
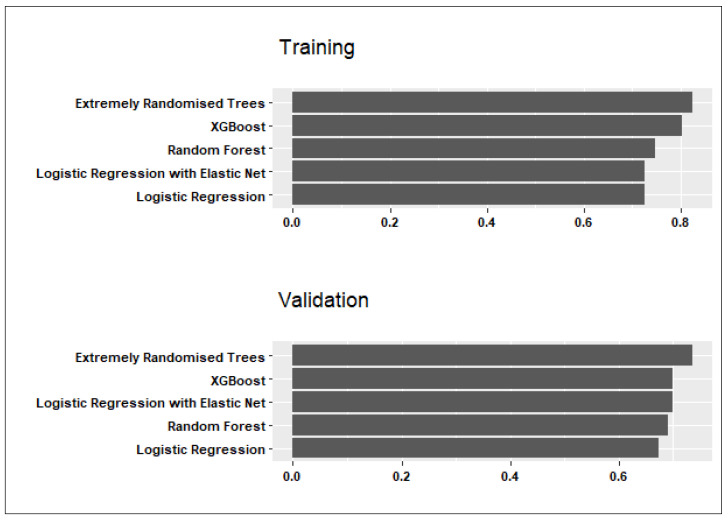
Comparison of candidate model specificity averaged across the training sets produced by three different data partitions.

The mean sensitivity and specificity of candidate models across all folds and data partitions were compared in both the training and validation sets. Figure 1 and Figure 2 show the candidate models’ performance averaged across the three data partitions. Below is a summary of the findings and conclusions:

Considering both the training and validation sets in Figure 1, the Random Forest model exhibited the highest sensitivity to PPH cases, with average values of 0.79 in training and 0.71 in validation. The average specificity to the controls was similar (0.76 in training; 0.70 in validation). On average, the penalised logistic regression exhibited slightly lower sensitivity to PPH cases than the logistic regression without an elastic-net penalty (0.72 in training and 0.69 in validation, compared to 0.73 in training and 0.73 in validation). 

No L1 penalty terms were applied to any features in the penalised logistic regression model over cross-validation in all data partitions. All features were retained in the penalised model, subject to a small L2 penalty constraining the regression coefficient size. The penalised logistic regression hyperparameters that maximised the ROC for each data partition are summarised in Table 1.

On average, the penalised logistic regression exhibited higher specificity to controls than the logistic regression without an elastic-net penalty (0.76 in training and 0.72 in validation, compared to 0.69 in both training and validation). Considering the penalised logistic regression hyperparameters summarised in Table 1, constrained regression coefficients may support the model’s specificity to the controls but not its sensitivity to PPH cases.

Although the Extremely Randomised Tree model had the highest specificity values of 0.81 in training and 0.74 in validation (see Figure 2), the average sensitivity to predicting PPH cases was somewhat lower than in the Random Forest model (0.74 in training and 0.70 in validation). 

Considering these results, the Random Forest model was selected as the final predictive model for PPH due to its good average sensitivity to PPH cases and comparable specificity to the controls in training and validation. The Random Forest hyperparameters that maximised the ROC for each data partition are summarised in Table 2.

**Figure 3:**
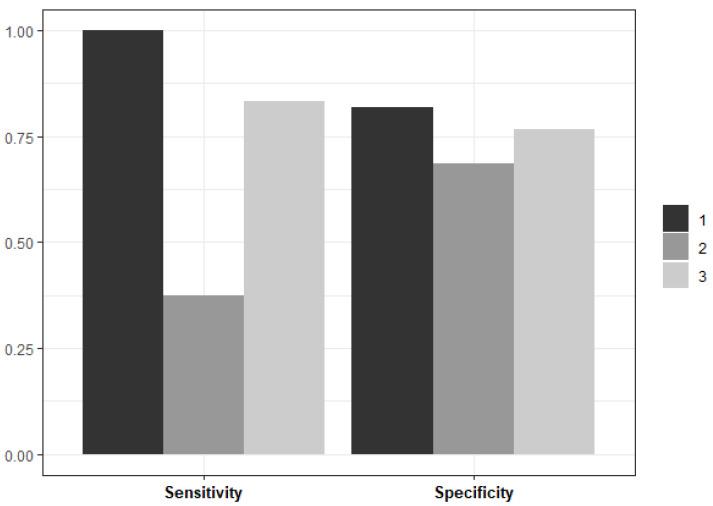
Summary of the final model’s performance on unseen data, averaged across the three data partitions.

The number of random features considered at each split was consistent across the data partitions (*n* = 2). However, there were some differences in the minimum size of the terminal nodes, with a minimum size of 20 in two data partitions and a minimum size of 25 in the remaining partition. The Random Forest model performance metrics on unseen test data, averaged across three data partitions, are plotted in Figure 3. 

In two out of the three data partitions, the Random Forest model performed well on the unseen data—both in terms of its sensitivity to PPH cases and its specificity to the controls. In Partition 2, the Random Forest model shows poor sensitivity to PPH cases compared to Partitions 1 and 2. However, the model’s specificity to the controls is similar across all the partitions. Although there are performance differences across the data partitions, the Random Forest model provides the best balance between sensitivity and specificity. The average sensitivity across all the partitions is 0.81, and the average specificity is 0.71. 

The average misclassification rate (12.19%) was calculated across the data partitions to provide a practical representation of the model’s predictive performance. Most misclassifications correspond to controls misclassified as PPH cases. This corresponds with the core objective of developing a predictive model for PPH: to identify high-risk women for targeted interventions.

After identifying the best predictive model for PPH, the next objective was to rank the importance of features in predicting PPH. Relative feature importance was estimated using the caret package by applying the final models to the test data for each data partition [28]. For Random Forest models, feature importance was determined using Gini impurity. This metric considers how many times a feature is used to create a partition (weighted by the number of bootstrap samples including that partition), then averages the decreases in Gini impurity resulting from splitting on that feature over all trees in the ensemble [29]. Estimates are scaled between 0 and 100 within each partition for comparison.

**Figure 4:**
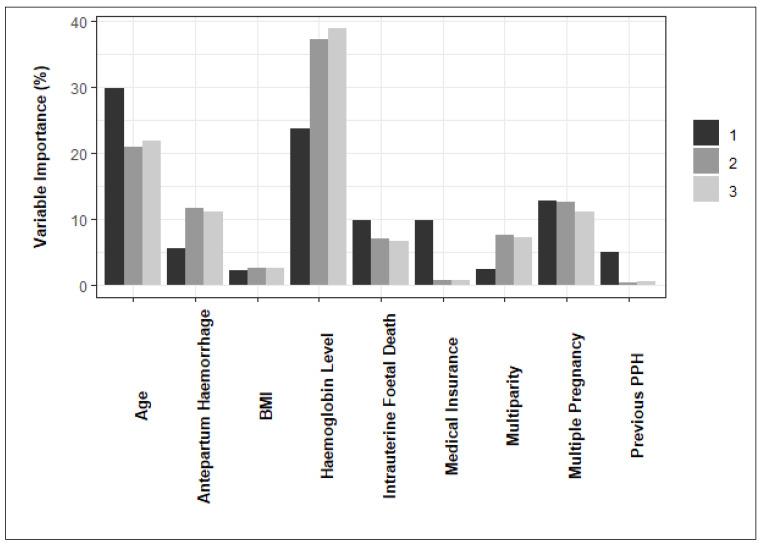
Ranking the importance of features in predicting PPH.

The variable importance estimates obtained from the final model for each data partition are summarised in Figure 4. According to the figure, haemoglobin level and maternal age are influential features in predicting PPH across all the partitions. 

Maternal age has a higher relative importance in the first data partition than in the second and third partitions. On the other hand, haemoglobin level during labour has a lower relative importance in the first data partition than in the second and third partitions. The importance of maternal BMI, intrauterine foetal death, and multiple pregnancy is lower but more consistent across the data partitions.

Although there are differences in relative importance across the data partitions, it is evident that maternal age and haemoglobin level have a high relative importance as predictors of PPH across all the partitions. Intrauterine foetal death and multiple pregnancy show a modest and consistent relative importance across the folds. 

## 4. Discussion

The research evaluated various predictive models—logistic regression, logistic regression using an elastic net, Random Forests, Extremely Randomised Trees, and XGBoost’s gradient-boosted trees—for predicting postpartum haemorrhage (PPH). 

The logistic regression model with all features had a lower sensitivity to PPH cases but a greater specificity to the controls. As only a small L2 penalty was applied to constrain the regression coefficients, this difference in performance may reflect the small feature set of early risk factors considered in this analysis. If all features are needed to model the variability in a target response, penalised logistic regression may not improve predictive performance.

The Random Forest model emerged as the most effective classifier for PPH across all folds and data partitions. Upon assessment with unseen test data, this model demonstrated impressive performance across the three data partitions. It achieved an average sensitivity of 80.7% in identifying PPH cases and a specificity of 71.3% in recognising controls, and it maintained an average misclassification rate of 12.19%.

Previous comparative studies by Venkatesh et al. [17] demonstrated that tree-based ensemble techniques, such as gradient-boosted trees with XGBoost and Random Forests, outperformed statistical models like logistic regression and lasso regression in predicting PPH. Similar findings were observed in other studies comparing various machine learning techniques in clinical predictive modelling for diseases, where tree-based ensemble techniques showed excellent predictive performance compared to neural networks, Support Vector Machines, and Bayesian approaches [30,31]. 

Machine learning techniques offer greater flexibility than statistical models, which appears necessary in this application to achieve the required separability between PPH cases and controls for good predictive performance. For medical professionals who intend to apply predictive modelling techniques to their own research, this flexibility may provide a practical advantage over statistical models, which require features and interactions to be prespecified.

The variable importance in the best-performing predictive model differed across the data partitions. However, despite these differences, it was possible to identify clear trends in relative importance regardless of the data partition. Maternal age and haemoglobin level have a high relative importance as predictors of PPH across all the partitions. 

This analysis highlights the value of considering the relative importance of predictors across multiple data partitions (see Figure 4). For example, if only Partition 1 were considered, medical insurance would be identified as a feature of similar relative importance to multiple pregnancy. However, in Partitions 2 and 3, medical insurance is an unimportant predictor of PPH relative to the other features. This insight may be relevant for medical professionals who would like to obtain a stable, generalisable estimate of variable importance from predictive models within their own research.

The variable importance of the final model did not completely align with the expectations based on the original study by Bazirete et al. [11] and the other literature reviewed at the beginning of this study. Bazirete et al. [11] found pre-labour haemorrhage and having no medical insurance to be significantly associated with PPH based on the *p*-values (*p* < 0.05) associated with the estimated risk ratios. Maternal age, haemoglobin level during labour, BMI, and multiparity were also found to be statistically significant in the original study. Mesfin et al. [12] identified a maternal age of 35 years and older as a significant risk factor of PPH when considering specific maternal age categories. However, features such as intrauterine foetal death and multiple pregnancy, which were highly significant in Bazirete et al. [11], had lower relative importance in the final model. A previous history of PPH, although identified as a significant risk factor for PPH by Bazirete et al. [11], was not considered an important predictor in the final model. 

The discrepancies observed in the variable importance between this analysis, Bazirete et al.’s analysis, [11] and the other literature reviewed may be attributed to the use of different predictive modelling techniques. Medical practitioners may benefit from considering several candidate models and estimating variable importance from the best-performing model. Estimating variable importance at an optimally selected classification threshold may provide medical practitioners with greater flexibility. 

The discrepancies observed in variable importance may be attributed to the use of different research methodologies and statistical models. Bazirete et al. [11] performed a Poisson regression, and the dataset was produced by an observational case–control study. Mesfin et al. [12] performed a logistic regression, and the dataset was produced by an institution-based cross-sectional study. Probability threshold selection for the classification of PPH cases was performed in this analysis, but not by Mesfin et al. [12]. This highlights the impact of variable importance from predictive models within their own research.

## 5. Conclusions

The objective of this study was to develop an accurate predictive model for postpartum haemorrhage (PPH) using early risk factors and to rank the most significant predictors of PPH. 

The dataset consisted of 430 observations from a previous study in Rwanda. Models, including logistic regression, elastic-net-regularised logistic regression, Random Forests, Extremely Randomised Trees, and Extreme Gradient Boosting, were trained and evaluated using cross-validation in three different train–test data partitions. 

The logistic regression model with all features had a lower average sensitivity to PPH cases than the penalised logistic regression model across all folds and data partitions. This performance difference may reflect the small feature set of early risk factors considered in this analysis. When deciding whether to implement penalised logistic regression, medical practitioners should be guided by the characteristics of their dataset—especially by the number of features.

The Random Forest model performed the best, demonstrating a good average sensitivity and specificity across all the folds and data partitions. Differences in classification performance were observed across the different data partitions. Considering classification performance across data partitions may therefore help identify a predictive model with more stable and generalisable performance.

This analysis highlights the tree-based ensemble technique of a Random Forest as a promising method for medical practitioners to apply in their own research. Its flexibility provides a practical advantage over statistical models for medical practitioners who intend to implement predictive modelling techniques in their own research. The transparency of the Random Forest partitioning process makes predictions from this technique more readily interpretable for medical practitioners than in many other machine learning techniques.

The most important predictors of PPH identified by the model were maternal age and haemoglobin level during labour. This was observed across all the data partitions, despite a higher relative importance for maternal age and a lower relative importance for haemoglobin level in the first partition. The importance of maternal BMI, intrauterine foetal death, and multiple pregnancy was lower but more consistent across the data partitions. The variations in findings regarding risk factors underline the value of considering the relative importance of predictors across multiple data partitions. Medical practitioners who wish to obtain a stable, generalisable estimate of variable importance from predictive models within their own research may wish to avoid considering a single data partition. 

The analysis has limitations, including a narrow focus on variables before and at labour admission as well as a small dataset, limiting generalisability. Given the small dataset, certain variables with low prevalences could not be considered, including uterine anomaly, uterine surgery, gestational diabetes mellitus, polyhydramnios, anticoagulant medication, and severe preeclampsia. The relationships between these low-prevalence variables and PPH cases would be best explored outside of a predictive modelling context.

To address these limitations, it is recommended to validate the findings with larger datasets and diverse populations. Validation using data from different time periods and conducting a cross-sectional study using medical records would enhance reliability. Furthermore, incorporating more intrapartum and immediate postpartum features should be considered for improved predictive performance, requiring further analysis and comparison of modelling techniques. 

Validating the findings in different settings and populations, including additional features, would strengthen the reliability and applicability of the developed predictive model for PPH. This validation is especially relevant given the lack of consensus in global clinical practice regarding the definition of PPH [3]. 

A validated predictive model estimating a patient’s risk of PPH based on early risk factors could form part of a clinical decision support system (CDSS) to support the accurate identification of women at higher risk of PPH. Receiving targeted information and recommendations at the point of care may help healthcare professionals navigate the complex factors influencing the detection and management of PPH in hospital settings for improved healthcare delivery.

## Figures and Tables

**Table 1:** Penalised logistic regression hyperparameters that maximised ROC for each data partition.

Data Partition	L1 Penalty Hyperparameter	L2 Penalty Hyperparameter
1	0	0.26
2	0	0.03
3	0	0.14

**Table 2:** Random Forest hyperparameters that maximised ROC for each data partition.

Data Partition	Number of Random Features	Minimum Terminal Node Size
1	2	20
2	2	25
3	2	20

## Data Availability

The dataset used for analysis is available in the paper by Bazirete et al. (2022) [11].
